# Pulmonary infection with *Aeromonas dhakensis* in a patient with acute T lymphoblastic leukemia: a case report and review of the literature

**DOI:** 10.3389/fmed.2024.1357714

**Published:** 2024-04-17

**Authors:** Chaoyang Wang, Nan Wei, Moyuan Zhang, Xiaoju Zhang

**Affiliations:** ^1^Department of Respiratory and Critical Care Medicine, Zhengzhou University People's Hospital, Henan Provincial People's Hospital, Zhengzhou, China; ^2^Xinxiang Medical University, Xinxiang, China; ^3^Department of Respiratory and Critical Care Medicine, Henan Provincial People's Hospital, Zhengzhou, China

**Keywords:** *Aeromonas dhakensis*, *Aeromonas*, pulmonary infection, mNGS, antibiotic

## Abstract

**Background:**

*Aeromonas dhakensis* is a gram-negative bacterium. In recent years, *Aeromonas dhakensis* has gradually attracted increasing attention due to its strong virulence and poor prognosis. Clinical reports of pulmonary infection caused by *Aeromonas dhakensis* are rare.

**Case presentation:**

A patient with acute T lymphoblastic leukemia experienced myelosuppression after chemotherapy, developed a secondary pulmonary infection with *Aeromonas dhakensis* and was hospitalized due to fever. The patient underwent testing for inflammatory markers, chest imaging, blood culture, bronchoalveolar lavage, pleural drainage, and metagenomic next-generation sequencing of alveolar lavage fluid and pleural fluid to obtain evidence of *Aeromonas dhakensis* infection, and was treated with four generations of cephalosporin combined with fluoroquinolone antibiotics. The patient’s condition significantly improved.

**Discussion:**

Among pulmonary infectious pathogens, *Aeromonas dhakensis* is relatively rare. Once an *Aeromonas* strain is cultured in the clinical work, pathogenic sequencing should be performed on the detected samples for early accurate diagnosis and effective anti-infection treatment.

## 1 Introduction

*Aeromonas dhakensis* is an emerging opportunistic pathogen. It was first found in the feces of children with diarrhea in Dhaka, Bangladesh, and described in 2002 (1). It is present in polluted water, fish and food. It often occurs in people with poor immune function, malignant tumors or biliary tract infections and can also be seen in healthy people who are in contact with polluted water. *Aeromonas dhakensis* can cause severe infection in the gastrointestinal tract, skin and soft tissues, even throughout the global body. Due to the complex classification of *Aeromonas*, routine laboratory tests cannot accurately identify *Aeromonas* species, and the epidemiology of *Aeromonas dhakensis* is underestimated in the clinic (2). Currently, *Aeromonas dhakensis* is considered the second most common pathogen of *Aeromonas* species and the most virulent (3–5). We report a case of pulmonary infection with *Aeromonas dhakensis* secondary to chemotherapy in a patient with acute T lymphoblastic leukemia. This infection was cured with standard antibiotics. This paper also reviews the treatment and prognosis of pulmonary infection with *Aeromonas dhakensis* and provides experience for clinicians in diagnosing and treating this disease.

## 2 Case report

An 18-year-old female patient was diagnosed with acute T-lymphoblastic leukemia more than 8 months earlier. She was started on vincristine, daunorubicin, cyclophosphamide, prednisone, and asparaginase (VDCLP) chemotherapy and leucocyte elevation therapy. Two days later, the patient developed fever, with a fluctuating body temperature and a highest temperature of 39°C. She was referred to our hospital the next day.

The admission test results were as follows: hemoglobin, 93 g/L (reference range: 115–150 g/L); platelet count, 74 × 10^9^/L (reference range: 125–350 × 10^9^/L); white blood cell count, 0.06 × 10^9^/L (reference range: 3.5–9.5 × 10^9^/L); neutrophils, not detected (reference range: 1.8–6.3 × 10^9^/L); lymphocytes, not detected (reference range: 1.1–3.2 × 10^9^/L); C-reactive protein (CRP), 25.32 mg/L (reference range: 0–10 mg/L); procalcitonin (PCT), 15 ng/L (reference range: < 0.05 ng/L); serum galactomannan (GM) test, negative; fungal detection (G) test, 86.2 pg/mL (reference range: < 60 pg/mL: negative, > 100 pg/mL: positive); cytokines: interleukin-6: 29.73 pg/mL (reference range: ≤5.4 pg/mL); interleukin-4: 9.03 pg/mL (reference range: ≤8.56 pg/mL); interferon-α: 49.3 pg/mL (reference range: ≤23.1 pg/mL); Lymphocyte immunoassay: CD19 + B lymphocytes: 0.27% (reference range: 6.58–24.52%); CD3 + T lymphocytes: 90.47% (reference range: 55.62–84.84%); CD3+/CD8+ toxic T lymphocytes: 49.69% (reference range: 13.27–40.63%); (CD3-CD16+/56+) NK cells: 4.31% (reference range: 5.15–27.08%); absolute number of lymphocytes: 37.6/μL (reference range: 1530–3,700/μL); absolute number of total T lymphocytes: 33.6/μL (reference range: 955–2,860/μL); absolute number of CD4+ helper T lymphocytes: 11.9/μL (reference range: 550–1,440/μL); absolute number of CD8+ toxic T lymphocytes: 17.3/μL (reference range: 320–1,250/μL); absolute number of B lymphocytes: 0.101/μL (reference range: 90–560/μL); and absolute number of NK cells: 1.72/μL (reference range: 150–1,100/μL). Acid-fast staining of the sputum smear was negative; fungal examination of the sputum smear was negative. Gram staining and microscopic examination of the sputum smear showed gram-positive cocci and gram-negative bacilli. Combined with the fever symptoms and laboratory results, the patient’s immune status was low at the time. Considering the diagnosis of “bone marrow suppression period after chemotherapy and coinfection,” anti-infection treatment made up of Biapenem 300 mg q8h as an intravenous infusion, vancomycin 0.5 g q8h as an intravenous infusion combined with posaconazole oral suspension 200 mg tid po was given for 6 days.

**Figure 1:**
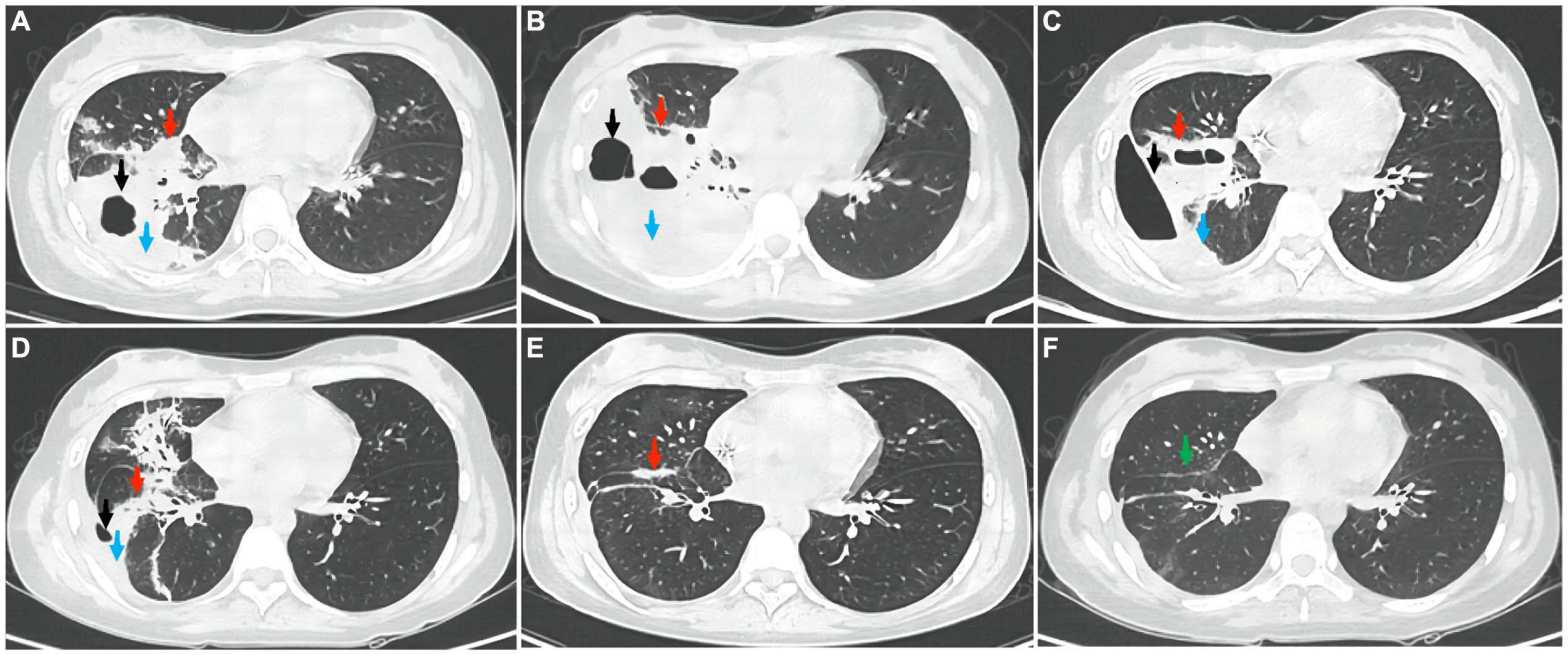
The picture shows the changes in the chest CT images during treatment. **(A)** the first CT scan in the hospital taken on 29 June 2023. The patient underwent a CT scan of the chest to observe the therapeutic effect on **(B)** 12 July 2023 and **(C)** 31 July 2023. Outpatient re-examinations using a repeat CT scan on **(D)** 25 August 2023, **(E)** 3 October 2023, and **(F)** 14 November 2023. The red arrow represents the inflammatory lesion, the black arrow shows the cavity, the blue arrow indicates fluid accumulation, and the green arrow represents residual striped shadows.

However, she still had fever and developed right chest pain on the 7th day after admission. Auscultation of the right lung indicated reduced breathing sounds. Complete chest computed tomography (CT) showed streaks and clumps of high-density shadows in the middle and lower lobes of the right lung with cavity formation and a small amount of fluid in the right chest cavity and interlobar fissure (Figure 1A). Blood in the sputum appeared on the 8th day. Blood examination showed that the white blood cell count was 6.24 × 10^9^/L, the neutrophil count was 5.98 × 10^9^/L, the lymphocyte count was 0.07 × 10^9^/L, CRP was 152.89 mg/L, and PCT was 1.96 ng/mL. The BD PhoenixTM Automated Microbiology System (BD Diagnostic Systems, Sparks, United States) was used for bacterial identification and drug sensitivity tests. Blood culture showed the growth of *Aeromonas hydrophila*. The drug sensitivity test results were as follows (Table 1): *Aeromonas hydrophila* was sensitive to amikacin, aztreonam, cefepime, cefotaxime, ceftazidime, chloramphenicol, ciprofloxacin, gentamicin, imipenem, levofloxacin, meropenem, piperacillin/tazobactam, and the compound sulfamide but resistant to tetracycline. Therefore, the antibiotic regimen was adjusted to Ceftazidime 2 g q8h as an intravenous infusion combined with moxifloxacin 0.4 g qd as an intravenous infusion for 13 days.

**Table 1:** Antibiotic susceptibility test for *Aeromonas dhakensis.*

Antibiotic	MIC (ug/mL)	Sensitivity
Amikacin	≤8	Sensitive
Aztreonam	≤2	Sensitive
Cefepime	≤2	Sensitive
Cefotaxime	≤1	Sensitive
Ceftazidime	≤1	Sensitive
Chloramphenicol	≤4	Sensitive
Ciprofloxacin	≤0.5	Sensitive
Gentamicin	4	Sensitive
Imipenem	≤1	Sensitive
Levofloxacin	≤1	Sensitive
Meropenem	≤1	Sensitive
Piperacillin/tazobactam	≤4/4	Sensitive
Tetracycline	>8	Resistant
Compound sulfamide	≤0.5/9.5	Sensitive

**Figure 2:**
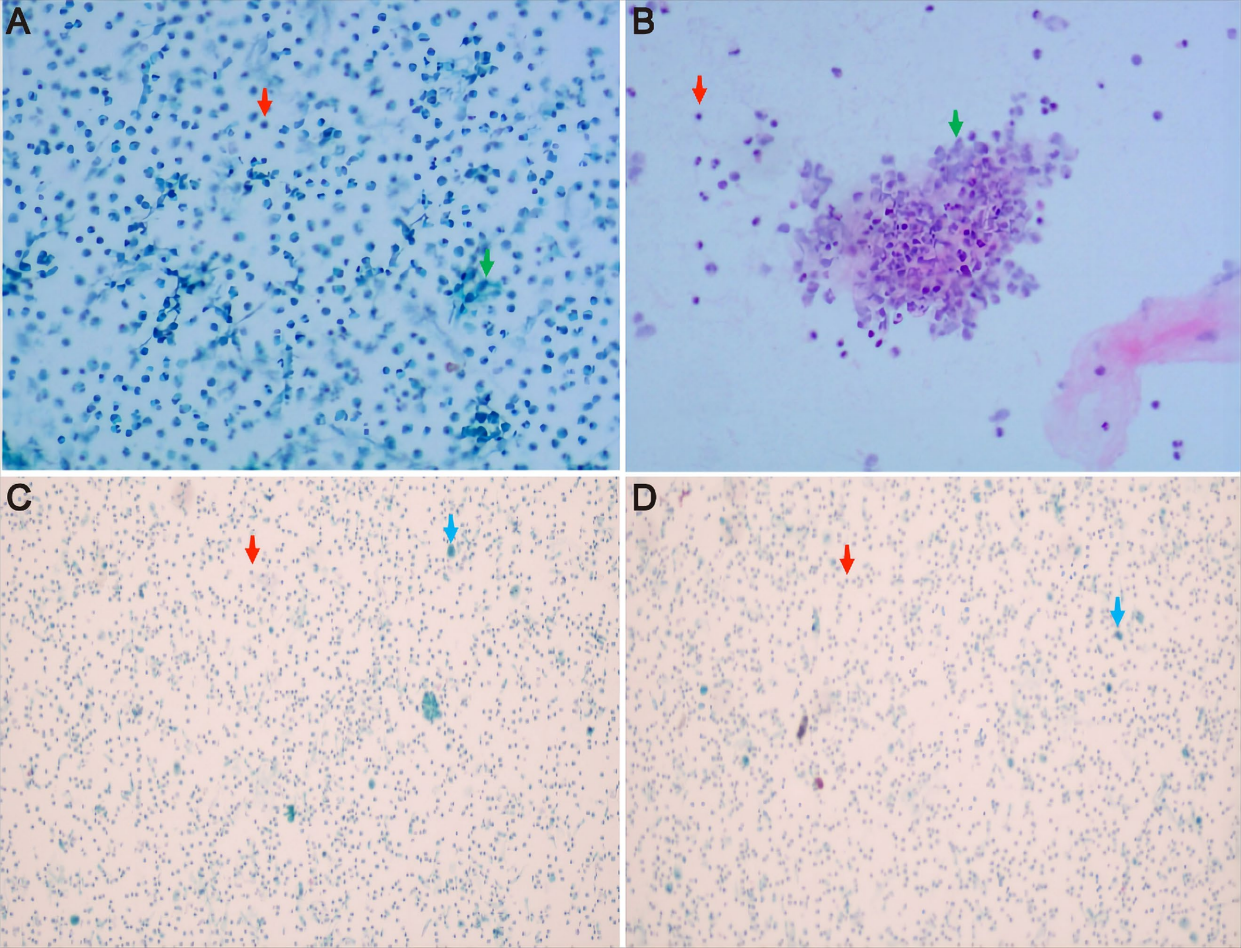
Cytological results of pleural fluid and alveolar lavage fluid by hematoxylin–eosin staining. **(A,B)** the pleural fluid Thinprep cytologic test showed inflammatory cells, with a small number of mesothelial cells and tissue cells, but no clear atypical tumor cells were observed. **(C,D)** alveolar lavage fluid under the microscope indicated ciliated columnar epithelial cells and inflammatory cells in the right middle lobe bronchial lavage fluid, but no clear atypical tumor cells were observed. The red arrow represents inflammatory cells, the green arrow shows mesothelial cells, and the blue arrow indicates ciliated columnar epithelial cells.

On the 20th day after admission, chest CT showed increased density in the middle and lower lobes of the right lung with stripy and clump shapes, a larger cavity area than before, and increased pleural effusion on the right side (Figure 1B). Considering the worsening of the patient’s hemoptysis symptoms, chest CT indicated progressive inflammation and pleural effusion. Therefore, bronchoalveolar lavage and pleural drainage were performed to help find unknown pathogens. Conventional testing of the pleural effusion revealed the following results: color, orange yellow; transparency, turbidity, visible solidification; Rivalta test, positive; and white blood cell count, 10,154 × 10^6^/L. The biochemistry of the pleural effusion showed the following: total protein 37.3 g/L, albumin 21.3 g/L, lactate dehydrogenase 6,537 U/L, and glucose 0.48 mmol/L. Ordinary bacterial culture of the pleural effusion detected no bacteria. Cytological staining of alveolar lavage fluid and pleural fluid via hematoxylin–eosin staining showed inflammatory cells (Figure 2). To identify potential pathogenic microorganisms, we performed metagenomic next-generation sequencing (mNGS) on the pleural effusion and bronchoalveolar lavage fluid. Pleural effusion mNGS indicated *Aeromonas dhakensis* (number of sequences: 43, identification confidence: 99%) (reference range: not found). Bronchoalveolar lavage fluid mNGS showed *Aeromonas dhakensis* (number of sequences: 101, identification confidence: 99%) (reference range: not found). According to the ABX POC-IT guidelines and published literature (6–12), *Aeromonas* is sensitive to fluoroquinolones and fourth-generation cephalosporins. Therefore, the antibiotic was adjusted to cefepime 2 g q8h as an intravenous infusion combined with levofloxacin 0.5 g qd as an intravenous infusion.

On the 38th day after admission, her body temperature returned to normal, and her symptoms of hemoptysis disappeared. Blood examination revealed a white blood cell count of 6.15 × 10^9^/L, a neutrophil count of 3.55 × 10^9^/L, a lymphocyte count of 1.81 × 10^9^/L, CRP of 28.85 mg/L, and PCT of <0.05 ng/mL. Chest CT showed that the cord-like and flaky-like high-density shadows in the middle and lower lobes of the right lung were reduced in scope, and the pleural effusion on the right side was less than that of the previous lesions (Figure 1C). Therefore, the patient was discharged home, and the original antibiotic regimen was continued. On the 63rd day of follow-up, her white blood cell count was 3.74 × 10^9^/L, her neutrophil count was 2.13 × 10^9^/L, her lymphocyte count was 1.05 × 10^9^/L, and her CRP level was 2.51 mg/L. Chest CT showed that the extent of inflammation and pleural effusion both significantly improved (Figure 1D). On the 101st day of follow-up, chest CT indicated that most of the inflammatory lesions had been absorbed (Figure 1E), so antibiotics were discontinued. On the 144th day of follow-up, chest CT showed that the inflammatory lesions had basically resolved, though a few traces remained (Figure 1F).

## 3 Discussion

*Aeromonas* infection often occurs in people who come into contact with contaminated water or food, people with liver and biliary diseases, people with blood diseases and people with weakened immunity. More than 30 phenotypes of *Aeromonas* have been reported (13), among which *Aeromonas dhakensis* can cause purulent lesions. According to the previously reported cases (Table 2), most were out-of-hospital infections, the disease progressed rapidly, the 14-day mortality rate was high, and the cause of death was mostly bacteremia or systemic infection. These reported cases were all misjudged in the early diagnostic process based on routine biochemical tests and were eventually confirmed by etiological sequencing (6, 14, 16, 18). Pulmonary infection caused by *Aeromonas dhakensis* has rarely been reported.

**Table 2:** Previous reports of *Aeromonas dhakensis* infection.

First author	Year	Location	Number	Age	Gender	Risk factor/comorbidity	Etiology	Position	Treatment	Mic	Prognosis
Chen et al. (3)	2013	China	7			Exposure to environmental water	rpoD and gyrB genes sequencing: *Aeromonas dhakensis*			/	
				33	Male	Flame burn		Multiple	Cefazolin, Gentamicin	/	Survival
				45	Male	Necrotizing fasciitis		Foot	Cefotaxime, Doxycycline	/	Survival
				47	Female	Trauma		Multiple	Nil	/	Survival
				62	Male	Necrotizing fasciitis		Foot	Cefazolin, Gentamicin	/	Survival
				67	Female	Surgical wound infection		Upper limb	Cephalexin	/	Survival
				74	Male	Necrotizing fasciitis		Lower limb	Ceftriaxone, Doxycycline	/	Survival
				75	Male	Cellulitis		Lower limb	Amoxicillin-clavulanic acid	/	Survival
Chang et al. (14)	2018	China	2				Blood or/and pus culture: *Aeromonas* *Hydrophila*. rpoD gene sequencing: *Aeromonas dhakensis*			/	
				65	Female	Dengue, Necrotizing Fasciitis		Bloodstream, left leg	Ceftazidime	/	Death
				75	Male	Dengue, Necrotizing Fasciitis		Bloodstream, lower limb	/	/	Death
Melo-Bolivar et al. (15)	2019	Australia	1	/	/	/	rpoB and gyrB genes sequencing: *Aeromonas dhakensis*	Necrotizing Fasciitis	/	/	Death
Huang et al. (16)	2019	China	1	29	Male	Snake headed fish, HBV	Blood culture and biochemical tests: *Aeromonas hydrophila*. The whole-genome sequencing: *Aeromonas dhakensis*	Bloodstream	Ceftriaxone Meropenem	≤1 ≤0.25	Death
Kitagawa et al. (6)	2019	Japan	4	57 to 90	2 males and 2 females	Solid cancer/Obstructive Biliary disease/Surgical History	Blood culture and biochemical tests: *Aeromonas hydrophila, Aeromonas caviae,* and *Aeromonas jandaei*. rpoD and gyrB genes sequencing: *Aeromonas dhakensis*	Bloodstream	/	/	3 survived and 1 died
Sun et al. (17)	2021	China	26	/	/	/	Blood culture and biochemical tests: *Aeromonas hydrophila*. gyrB gene sequencing: *Aeromonas dhakensis*	Bloodstream	/	/	12 died or treatment failed

The common pathogens of community-acquired pneumonia in patients with hematological malignancies were *fungi*, *Mycobacterium tuberculosis*, *Pseudomonas aeruginosa* and *Staphylococcus aureus*. Identification of pathogenic microorganisms occurs at least 24–48 h after admission, and empirical antibiotic treatment is one of the factors that leads to prolonged disease and then worsening infection (19, 20). *Aeromonas* is not a common opportunistic pathogen in patients with hematological malignancies. The patient in this case had no history of contact with fish or sewage, any history of travel, or any skin injuries before the onset of the disease. No significant therapeutic effect was achieved by the early empirical anti-infection treatment. After blood culture indicated the presence of *Aeromonas hydrophila*, we adjusted the antibiotic regimen to ceftazidime combined with moxifloxacin according to the drug sensitivity test results. Unfortunately, re-examination via chest CT indicated that the pulmonary infection had progressed. We wondered whether there was an undetected pathogen in the lungs or whether the pathogens were not fully covered by the original antibiotic regimen. To better identify the pathogen, the patient underwent bronchoalveolar lavage and pleural drainage, and the respiratory tract samples were subjected to mNGS by Vision Medicals (Guangzhou, China). Only *Aeromonas dhakensis* was detected in both bronchoalveolar lavage fluid and pleural effusion by mNGS, which was not consistent with the blood culture results. Traditional mNGS requires clinical presets to select DNA and/or RNA assay procedures, which may cause pathogen omission. We did mNGS with IDseq™ Ultra, which is a bidirectional enrichment technique (forward enrichment: multiple primers were designed to amplify the target pathogen; reverse enrichment: reducing the proportion of human cells or nucleic acids to the host) based on the combination of pathogen metagenomics and probe capture. It has the advantages of being comprehensive, sensitive and in depth. Some studies have shown that *Aeromonas dhakensis* can be misidentified as *Aeromonas hydrophila* by blood culture and subsequent phenotypic identification. According to the above data, the possibility of *Aeromonas hydrophila* and *Aeromonas dhakensis* coinfection cannot be ruled out. In addition, *Aeromonas* was not cultured from the pleural effusion, which may be due to differences in the *in vitro* and *in vivo* bacterial culture environments of the pleural effusion. Therefore, in clinical practice, when the anti-infective treatment regimen based on blood culture and corresponding drug sensitivity tests has not effectively improved the patient’s condition, it is particularly important to combine mNGS to further discover evidence of etiological infection to guide diagnosis and treatment. Both *Aeromonas dhakensis* and *Aeromonas hydrophila* are *Aeromonas* strains, but the former is more virulent. It is important to use antibiotics that can cover *Aeromonas dhakensis* and *Aeromonas hydrophila*. We consulted the ABX guidelines and found that *Aeromonas* was sensitive to aminoglycosides, fluoroquinolones, cabapenem, aztreonam, and third-and fourth-generation cephalosporins (11). Related studies have shown that second-and third-generation cephalosporins (cefuroxime, ceftriaxone, and ceftazidime) and carbapenem antibiotics (imipenem) have higher minimum inhibitory concentrations and drug resistance rates against *Aeromonas dhakensis* than against *Aeromonas hydrophila*, while both bacteria are sensitive to fourth-generation cephalosporins (cefepime) and fluoroquinolone antibiotics (levofloxacin) (3, 8, 9). With the overuse of antibiotics in agriculture, fish culture and clinical environments, the resistance of *Aeromonas* to drugs has increased in recent years, and the prevalence of multidrug-resistant *Aeromonas* has been on the rise (7, 17). Furthermore, pathogenic *Aeromonas* species are reported to be resistant to carbapenems due to the presence of the *CphA* gene, which encodes a metallo-β-lactamases that hydrolyzes carbapenems but not fourth-generation cephalosporins (9, 12, 21). Chromosomal AmpC β-lactamases which species-specifically distributes in *Aeromonas* strains and included AsbA1 (*Aeromonas jandaei*), CepH and CepS (*Aeromonas hydrophila*), CAV1 and MOX (*Aeromonas caviae*), TRU-1 (*Aeromonas enteropelogenes*), and AQU-1 (*Aeromonas dhakensis*) (9, 19, 22–26), can hydrolyze cephamycins and third-generation cephalosporins (27, 28). Based on the above data, we selected a fourth-generation cephalosporin (cefepime) combined with a fluoroquinolone drug (levofloxacin), and the patient’s symptoms and signs improved significantly. Compared with the deaths reported by Chang et al. and Huang et al. (14, 16), in which all patients received third-generation cephalosporin treatment immediately after admission but still died quickly, timely adjustment of the antibiotic to a fourth-generation cephalosporin in this case may have been critical to reduce the risk of death and improve the condition of the patient.

## 4 Conclusion

*Aeromonas dhakensis* is virulent and has potential drug resistance, which causes rapid progression of pulmonary infection. Because it is not common in the clinic, the diagnostic specificity is poor when the evidence is based only on the characteristics of symptoms and blood culture. Once the *Aeromonas* strain is cultured in the clinical work, the detected sample can be further subjected to mNGS for accurate diagnosis and improvement of the antibiotic regimen. Therefore, when specific antibiotics are selected for anti-infection treatment based on biological sample culture and the corresponding drug sensitivity tests, but the patient’s condition still does not improve much, adding mNGS may identify the etiology and guide the choice of anti-infection therapy.

## Data availability statement

The original contributions presented in the study are included in the article/supplementary material, further inquiries can be directed to the corresponding author.

## Ethics statement

The studies involving humans were approved by the Ethics Committee of Zhengzhou University People’s Hospital. The studies were conducted in accordance with the local legislation and institutional requirements. The participants provided their written informed consent to participate in this study. Written informed consent was obtained from the individual(s) for the publication of any potentially identifiable images or data included in this article.

## Author contributions

CW: Conceptualization, Data curation, Formal analysis, Writing – original draft. NW: Data curation, Investigation, Writing – review & editing. MZ: Data curation, Methodology, Writing – original draft. XZ: Conceptualization, Writing – review & editing.
